# Vitamin B-6 Supplementation Could Mediate Antioxidant Capacity by Reducing Plasma Homocysteine Concentration in Patients with Hepatocellular Carcinoma after Tumor Resection

**DOI:** 10.1155/2016/7658981

**Published:** 2016-03-09

**Authors:** Shao-Bin Cheng, Ping-Ting Lin, Hsiao-Tien Liu, Yi-Shan Peng, Shih-Chien Huang, Yi-Chia Huang

**Affiliations:** ^1^Division of General Surgery, Department of Surgery, Taichung Veterans General Hospital, Taichung 40705, Taiwan; ^2^School of Medicine, Chung Shan Medical University, Taichung 40201, Taiwan; ^3^School of Nutrition, Chung Shan Medical University, Taichung 40201, Taiwan; ^4^Department of Nutrition, Chung Shan Medical University Hospital, Taichung 40201, Taiwan

## Abstract

Vitamin B-6 has a strong antioxidative effect. It would be useful to determine whether vitamin B-6 supplementation had effects on antioxidant capacities in patients with hepatocellular carcinoma (HCC) who had recently undergone tumor resection. Thirty-three HCC patients were randomly assigned to either the placebo (*n* = 16) group or the vitamin B-6 50 mg/d (*n* = 17) group for 12 weeks. Plasma pyridoxal 5′-phosphate, homocysteine, indicators of oxidative stress, and antioxidant capacities were measured. Plasma homocysteine in the vitamin B-6 group was significantly decreased at week 12, while the level of trolox equivalent antioxidant capacity (TEAC) was significantly increased at the end of the intervention period. Vitamin B-6 supplementation had a significant reducing effect on the change of plasma homocysteine (*β* = −2.4, *p* = 0.02) but not on the change of TEAC level after adjusting for potential confounders. The change of plasma homocysteine was significantly associated with the change of TEAC after adjusting for potential confounders (*β* = −162.0, *p* = 0.03). Vitamin B-6 supplementation seemed to mediate antioxidant capacity via reducing plasma homocysteine rather than having a direct antioxidative effect in HCC patients who had recently undergone tumor resection. The clinical trial number is NCT01964001, ClinicalTrials.gov.

## 1. Introduction

The mortality rate of liver cancer has risen over the past decade and is now the 2nd most common cause of cancer death worldwide [1]. Liver cancer is also the 2nd cause of cancer-related death among men and women in Taiwan (Health Promotion Administration, Ministry of Health and Welfare, 2014). Hepatocellular carcinoma (HCC) is the most common type of liver cancer. In addition to the recognized risk factors of HCC, such as age, chronic hepatitis, cirrhosis, environmental toxins, and drugs, deficient vitamin B-6 status has been suggested to be a causative factor rather than a consequence of HCC development [2]. However, the mechanism by which low vitamin B-6 affects the oncogenesis and tumor progression of HCC has not been clearly established. The oncosuppressive functions of vitamin B-6 have been proposed to be possibly linked with the improvement of immune responses, the involvement of one-carbon metabolism, or the regulation of pyridoxal kinase and phosphatase [3]. However, the reduced effect of plasma homocysteine concentration by vitamin B-6 and the antioxidative property of vitamin B-6 might also play a role in the protection of HCC development but the mechanisms involved are not fully understood.

Vitamin B-6 has a strong antioxidative effect since its compounds have both the hydroxyl (–OH) and amine (–NH_2_) groups substitution on a pyridine ring which can directly react with the peroxy radicals and thereby scavenge radicals and lipid peroxidation [4–8]. The mechanism by which vitamin B-6 plays an indirect antioxidative role might involve the plasma pyridoxal 5′-phosphate (PLP, the physiological coenzyme form of vitamin B-6), which is an essential coenzyme in the transulfuration pathway for the conversion of homocysteine to cysteine. Inadequate plasma PLP, therefore, might cause homocysteine accumulation and further reduce cysteine generation. The oxidation of homocysteine can generate oxygen free radicals to enhance oxidative stress and diminish DNA methylation in critical tissues [9, 10]. Cysteine is an important substrate in the synthesis of glutathione (GSH) [11], which is a key regulator of intracellular redox and antioxidative reactions [12, 13]. A decrease in the activities of GSH-dependent antioxidant enzymes [i.e., glutathione peroxidase (GPx), glutathione* S*-transferase (GST)] may disrupt the balance between pro- and antioxidants, leading to cellular damage and cancer development [14]. Therefore, vitamin B-6 might protect against the development of HCC via its direct or indirect antioxidant property.

Previous studies indicated that plasma and liver tissue malondialdehyde (MDA) level (oxidative stress indicator) was higher and plasma antioxidant enzyme activities [i.e., superoxide dismutase (SOD), GPx] were lower in patients with HCC than in healthy controls [15–18]. Patients with HCC have been observed to have lower plasma PLP when compared to those of healthy controls [2, 16, 19]. If HCC patients are under increased plasma homocysteine and oxidative stress and decreased antioxidant capacities, this may possibly exhaust the use and metabolic turnover of plasma PLP and decrease hepatic PLP reserves. Since deficient vitamin B-6 status might be a causative factor for HCC development [2], it would be useful to determine whether vitamin B-6 supplementation would have a preventive effect in reducing plasma homocysteine or indirectly increasing GSH and its dependent antioxidant enzyme capacities in patients with HCC. The purpose of this study was to investigate whether vitamin B-6 supplementation had a significant effect on plasma homocysteine, oxidative stress, and antioxidant capacities in HCC patients following tumor resection.

## 2. Subjects and Methods

### 2.1. Patients

Consecutive patients who were diagnosed with primary HCC (International Classification of Diseases 9, code 155.0) and were scheduled to undergo tumor resection were invited to participate in this intervention study at the Division of General Surgery of Taichung Veterans General Hospital, Taiwan. Diagnosis and staging were confirmed by an oncologist and a pathologist. Patients were excluded if they were younger than 20 years or older than 80 years, were pregnant or lactating, were receiving chemotherapy, had a history of or current diagnosis of cardiovascular or renal diseases, or were taking any medication which could influence vitamin B-6 status. Patients taking vitamins or other nutritional supplements were asked to stop for at least 1 month before entering the study. Each subject signed an informed consent form before random assignment to the study groups. This study was approved by the Institutional Review Board of Taichung Veterans General Hospital.

### 2.2. Experimental Protocol

A minimal sample size of 14 patients in each group would allow the detection of significant changes of plasma homocysteine at 0.5 ± 0.6 *μ*mol/L after vitamin B-6 intervention with statistical power of 80% and a two-sided *α* level of less than 0.05.

Thirty-three patients were randomly assigned to either the placebo (*n* = 16) or vitamin B-6 50 mg/d group (*n* = 17) two weeks after they had undergone tumor resection and were blinded to their assigned groups. Intervention was administered for 12 weeks. The placebo (dextrin starch) (New Health Products Co., Ltd., Taipei, Taiwan) and vitamin B-6 capsules were commercially available preparations (Taiwan Veterans Pharmaceutical Co., Ltd., Chung-Li, Taiwan). Patients were instructed to take capsules daily and to refrain from using any other nutrient supplements during the study period. To ensure good compliance, investigators called every week to remind patients to take the capsules. Patients returned to the clinic to get additional supplements, at which time any unused capsules from the previous 4 weeks were returned and counted. Patients were taught to maintain their usual diets and activities during the study period.

The subjects' age, gender, height, weight, smoking and drinking habits, and use of medications were recorded. Subjects' body mass index (BMI, kg/m^2^) was calculated from their height and weight. Systolic and diastolic blood pressure were measured after a resting period of at least 5 min. Patients had their fasting blood drawn before the start of the intervention (week 0) and when they returned to the clinic every 4 weeks (week 4, week 8, and week 12). Venous blood samples were collected in Vacutainer tubes (Becton Dickinson, Rutherford, NJ) containing EDTA as an anticoagulant or no anticoagulant as required to determine hematological entities [i.e., albumin, creatinine, alanine aminotransferase (ALT) and aspartate aminotransferase, blood urea nitrogen, and alkaline phosphatase], plasma PLP, homocysteine and MDA concentrations, and antioxidant capacities [i.e., trolox equivalent antioxidant capacity (TEAC), GSH, oxidized GSH (GSSG), GPx, glutathione reductase (GR), and GST]. Blood samples were transported on ice and separated into plasma (or serum) and red blood cells within 1 h by speed centrifugation at 2500 rpm for 15 min. Samples were then frozen (−80°C) and stored until analysis.

Plasma PLP and homocysteine were determined by high performance liquid chromatography as previously described by Talwar et al. [20] and Araki and Sako [21], respectively. The interassay variability for plasma PLP and homocysteine was 4.4% (*n* = 8) and 3.7% (*n* = 7), respectively. Vitamin B-6 and homocysteine measurements were carried out under yellow light to prevent photodestruction. Plasma MDA was measured by thiobarbituric acid reactive substances at an excitation wavelength of 515 nm and an emission wavelength of 555 nm using a fluorescence spectrophotometer [22]. The TEAC level was measured according to a method described by Erel (2004) [23]. GSH, GSSG, and antioxidant enzyme activities, including activities of SOD, GPx, GR, and GST, were determined using GSH, GSSG, SOD, GPx, GR, and GST commercial kits (Cayman Chemical Company, Ann Arbor, MI, USA), respectively. All analyses were performed in duplicate.

### 2.3. Statistical Analyses

Data were analyzed using the SAS statistical software package (version 9.3; Statistical Analysis System Institute Inc., Cary, NC, USA). The Shapiro-Wilk test was performed to test the normal distribution. The changes of biochemical variables before and after vitamin B-6 supplementation were calculated as the value at week 12 minus the value at week 0 (Δ week 12 − week 0). Demographic characteristics and biochemical data of placebo and vitamin B-6 groups were compared to determine significant differences using Student's *t*-test or Mann-Whitney Rank Sum test at weeks 0, 4, 8, and 12. Chi-square or Fisher's exact tests were used for the analysis of categorical variables. One-way repeated measures analysis of variance was used to compare differences with values at weeks 0, 4, 8, and 12 within the group. Multiple linear regressions were used to assess the effect of vitamin B-6 supplementation (placebo group = 0, vitamin B-6 group = 1) on plasma homocysteine, oxidative stress, and antioxidant capacities after adjusting for age, gender, BMI, the baseline level (week 0) of creatinine and ALT, smoking and drinking habits, and HCC stage. Statistical results were considered to be significant at *p* < 0.05. Values presented in the text are means ± standard error (SE).

## 3. Results

Subjects' demographic and health characteristics are shown in Table 1. All patients had class A severity of liver disease according to the Child-Pugh classification. Twenty patients were in stage I and 13 patients were in stage II of HCC according to the TNM system. Twenty-four patients had tumor cells with poor differentiation while 2 and 7 patients had well or moderately differentiated tumor cells, respectively. Subjects' ages were between 30 and 79 years, with mean and medium ages of 58.8 and 61 years, respectively. The two groups had comparable age, gender, BMI, systolic and diastolic blood pressure, serum albumin and creatinine, cancer stage, and smoking and drinking habits.

Responses of clinical and biochemical measurements to placebo or vitamin B-6 supplements are shown in Table 2. The compliance of vitamin B-6 supplementation was achieved for at least 90% of all patients in the vitamin B-6 group. There were no significant differences with respect to all clinical and biochemical measurements between the placebo and vitamin B-6 groups at baseline (week 0). Mean plasma PLP level was adequate (PLP > 20 nmol/L) at week 0 for both groups. Plasma PLP significantly increased in response to vitamin B-6 supplementation in the vitamin B-6 group while plasma PLP remained stable throughout the intervention period in the placebo group. There were no significant changes in blood urea nitrogen, ALT and aspartate aminotransferase, alkaline phosphatase, MDA, activities of SOD, GPx, and GST during the intervention period for both groups. Plasma homocysteine and GSH concentrations, GSH/GSSG ratio, and activity of GR in the vitamin B-6 group significantly decreased by week 12, while total antioxidant capacity (i.e., TEAC level) significantly increased at the end of the intervention period.

Vitamin B-6 supplementation had no effect on the changes (Δ week 12 − week 0) of MDA, GSH, GSSG, and TEAC levels and the activities of SOD, GPx, GST, and GR (*p* > 0.05), but it significantly reduced (*β* = −2.4, standard error = 1.0, *p* = 0.02) plasma homocysteine after adjusting for age, gender, BMI, the baseline (week 0) levels of creatinine and ALT, smoking and drinking habits, and HCC stage. In order to determine whether the change of TEAC level was due to the change of plasma homocysteine concentration, multiple linear regression analysis was also used. The change of plasma homocysteine had a significant effect on the change of TEAC after adjusting for age, gender, BMI, the baseline (week 0) level of creatinine and ALT value, smoking and drinking habits, and HCC stage (*β* = −162.0, standard error = 67.2, *p* = 0.03).

## 4. Discussion

To the best of our knowledge, this is the first study to show that a large dose of vitamin B-6 (50 mg/d) given to HCC patients who had just undergone tumor resection for 12 weeks was effective in reducing plasma homocysteine concentration which could further improve the total antioxidant capacity (i.e., TEAC level).

Previous studies observed that patients with liver cirrhosis (a precursor of HCC) had hyperhomocysteinemia [24–26]. Plasma PLP is a coenzyme in the transsulfuration pathway of homocysteine metabolism, and thus plasma homocysteine concentration might be associated with vitamin B-6 status. In our previous animal study [27], mice fed a vitamin B-6 deficient diet had the highest homocysteine concentration in plasma and liver among other mice fed diets with adequate vitamin B-6 or supplemented with vitamin B-6. However, there are no data on the effect of vitamin B-6 supplementation on lowering fasting plasma homocysteine concentration in HCC patients who had just undergone tumor resection. In the present study, 50 mg of vitamin B-6 supplementation seemed to have a beneficial effect on reducing plasma homocysteine concentration at the 4th week of intervention and this phenomenon was maintained throughout the whole intervention period, and the effect of reduced homocysteine by vitamin B-6 supplementation was not confounded by potential confounders. In spite of the well-known relationship between high plasma homocysteine concentration and the risk of cardiovascular disease, high plasma homocysteine concentration has been proposed to be a better tumor marker for monitoring cancer patients during therapy and a sensitive marker for the detection of cancer recurrence [9]. Since the reduced homocysteine concentration by vitamin B-6 supplementation was effective in this study, it is reasonable to be cautiously optimistic that a lower rate of HCC recurrence via reduced homocysteine concentration can be expected in our patients who had just undergone tumor resection. Follow-up studies should be conducted to monitor the effect of reduced homocysteine by vitamin B-6 supplementation on the recurrence of HCC.

Increased oxidative stress and decreased antioxidant enzyme activities are thought to be one of the pathogenic mechanisms of HCC [13, 15, 16]. Vitamin B-6 has been found to prevent oxygen radical generation and lipid peroxidation caused by H_2_O_2_ in U937 monocytes [6] and endothelial cells [28]. The levels of total and nonenzymatic superoxide scavenger activity and antioxidant potential in the kidney tissue of vitamin B-6 deficient rats were significantly lower than those of the control rats [8]. Although vitamin B-6 has a potential antioxidant property, there is a lack of evidence linking vitamin B-6 status as wells as oxidative stress and antioxidant capacities with risk of HCC. Previous animal studies indicated that vitamin B-6 deficiency caused an increase in oxidative stress and a decrease in the antioxidant defense system in the liver tissue of rats and mice [27, 29]; vitamin B-6 supplementation had a protective effect against the increased oxidative stress markers (i.e., MDA and advanced oxidation protein products) induced in hyperhomocysteinemic rats [7]. In a human study, the decreases in plasma and erythrocyte PLP concentrations increased oxidative stress (i.e., MDA level) and decreased antioxidant enzyme activities in HCC patients [16]. MDA is a tumor promoter and cocarcinogenic agent due to its high cytotoxicity and inhibitory action on protective enzymes [15]. Although plasma PLP concentration was significantly increased in our HCC patients who received vitamin B-6 supplementation, the MDA level was only slightly but not significantly decreased. It is worth noting that our HCC patients did not have excessive MDA levels when compared to the levels in stages I and II HCC patients reported in a previous study [16]. Our HCC patients had recently undergone tumor resection which might have reduced their oxidative stress; therefore, even an increased plasma PLP concentration might not have further reduced the MDA level. In addition to MDA, the levels of other oxidative stress indicators, such as protein carbonyl and 8-hydroxydeoxyguanosine, were found to be elevated in HCC patients when compared to healthy controls [30, 31]. However, we did not measure levels of other oxidative stress indicators in our HCC patients, and thus the changes of other oxidative stress indicators before and after vitamin B-6 supplementation could not be compared. In contrast to the unchanged MDA level, TEAC level was significantly increased by vitamin B-6 supplementation at week 12. Surprisingly, the effect of vitamin B-6 supplementation on TEAC level disappeared after adjusting for potential confounders; vitamin B-6 supplementation seemed to have no direct role on total antioxidant capacity. Since high plasma homocysteine concentration would increase oxidative stress through homocysteine oxidation [9, 10], we postulated that reduced plasma homocysteine concentration by vitamin B-6 supplementation in our HCC patients may diminish the production of oxygen free radicals and thus provide a protective mechanism which would combat oxidative stress. We then assessed the association between the change of plasma homocysteine and TEAC level and found that the reduced plasma homocysteine concentration after 12 weeks of vitamin B-6 supplementation was a significant factor in improving total antioxidant capacity in our HCC patients. This suggests that vitamin B-6 supplementation might mediate antioxidant capacity by reducing plasma homocysteine rather than by directly having an antioxidative protection effect in HCC patients who had just undergone tumor resection.

The GSH and its dependent enzymes play a fundamental role in cellular defense against reactive free radicals and other oxidant species [12, 32]. GPx is a selenoprotein which can reduce hydroperoxides and H_2_O_2_ but oxidizes GSH to become GSSG, and GR then reduces GSSG to GSH. A previous study indicated there was a decrease in the GSH level and increases in the GSSG level and GR activity in 54 HCC patients compared with those of 57 controls [17]. The increased GPx and GR activities might be due to enhanced oxidative stress in HCC patients. We postulated that since vitamin B-6 is indirectly involved in GSH synthesis, vitamin B-6 supplementation might have an influence on the performance of the GSH-dependent antioxidant system. However, we could not explain why the GSH/GSSG ratio and GR activity decreased while the GSSG level and GPx activity remained constant in the vitamin B-6 supplemented group. It is likely that a large dose of vitamin B-6 supplementation might directly reduce plasma homocysteine which would further increase antioxidant capacity to balance the oxidant and antioxidant status rather than being indirectly involved in the GSH-related antioxidant defense system in HCC patients who had just undergone tumor resection. On the other hand, we could not rule out the possibility that the reduced plasma GSH synthesis might just be due to the redistribution from plasma to erythrocytes during vitamin B-6 supplementation. Unfortunately, erythrocyte GSH concentration was not analyzed in this study, the distribution of plasma and erythrocyte GSH during vitamin B-6 supplementation could not be discussed further. In addition to cysteine, glutamate and glycine are both involved in GSH synthesis, probably playing more dominant roles than vitamin B-6 in the regulation of GSH and its dependent enzyme activity. Further study is warranted to study the interrelationships among cysteine, glutamate, glycine, GSH, and its related enzyme activities in HCC patients.

There were some limitations in this study. First, we calculated the sample size to meet the statistical power prior to the start of the intervention; however, including a greater number of subjects in each group might increase the statistical power thereby possibly revealing a more significant effect of vitamin B-6 supplementation on plasma homocysteine, oxidative stress, and antioxidant capacities. Second, this study was originally designed as a double-blind study; however, the blinding of researchers failed due to an oversight and therefore a single-blind approach was employed instead. Third, follow-up was not performed in this study and, therefore, the long-term effect of vitamin B-6 supplementation on the recurrence of HCC and clinical outcomes could not be investigated.

## 5. Conclusions

In conclusion, 50 mg/d of vitamin B-6 supplementation significantly reduced plasma homocysteine concentration, which further improved total antioxidant capacity in HCC patients who had just undergone tumor resection. Vitamin B-6 supplementation seemed to mediate the antioxidant capacity by reducing plasma homocysteine rather than by having a direct antioxidative effect in HCC patients.

## Figures and Tables

**Table 1:** Demographic and health characteristics of subjects^1^.

	Placebo (*n* = 16)	Vitamin B-6 (*n* = 17)
Age (y)	61.0 ± 2.7	56.7 ± 3.6
Sex (male/female)	9/7	13/4
Body mass index (kg/m^2^)	25.1 ± 1.0	24.6 ± 0.8
Blood pressure (mmHg)		
Systolic	130.4 ± 5.1	125.0 ± 3.3
Diastolic	73.6 ± 3.0	74.9 ± 3.0
Serum albumin (g/dL)	4.1 ± 0.1	4.4 ± 0.1
Serum creatinine (mg/dL)	0.8 ± 0.1	0.9 ± 0.1
Cancer stage (*n*, %)		
Stage I	9 (56.3%)	11 (64.8%)
Stage II	7 (43.8%)	6 (35.3%)
Histological grading (*n*, %)		
Well differentiated (I)	1 (6.3%)	1 (5.9%)
Moderate differentiated (II)	3 (18.8%)	4 (23.5%)
Poor differentiated (III)	12 (75%)	12 (70.6%)
Smoking (*n*, %)		
Yes	4 (25%)	4 (23.5%)
No	12 (75%)	13 (76.5%)
Drinking (*n*, %)		
Yes	3 (18.8%)	2 (11.8%)
No	13 (81.3%)	15 (88.2%)

^1^Values are means ± standard error of mean (SE).

**Table 2:** Responses of biochemical measurements to placebo or vitamin B-6 supplementation during and after the intervention period^1^.

Biochemical measurements	Placebo (*n* = 16)				Vitamin B-6 (*n* = 17)			
	wk 0	wk 4	wk 8	wk 12	wk 0	wk 4	wk 8	wk 12
PLP (nmol/L)	90.6 ± 25.0	96.9 ± 31.0	58.3 ± 7.8	80.3 ± 18.5	91.2 ± 30.8^a^	347.4 ± 77.3^b,*∗∗*^	337.9 ± 46.5^b,†^	354.7 ± 61.3^b,††^
Liver function								
BUN (mg/dL)	15.4 ± 1.3	14.8 ± 1.1	15.9 ± 1.4	15.2 ± 1.0	16.6 ± 1.5	15.4 ± 1.4	15.1 ± 1.3	15.4 ± 1.1
ALT (U/L)	48.6 ± 9.7	50.5 ± 11.0	53.8 ± 14.0	57.5 ± 16.1	48.6 ± 7.1	40.3 ± 4.4	40.5 ± 5.1	49.9 ± 12.4
AST (U/L)	37.1 ± 6.7	36.9 ± 6.8	40.8 ± 9.2	44.1 ± 9.0	32.0 ± 3.5	34.2 ± 4.2	35.4 ± 3.4	38.5 ± 4.6
ALP (U/L)	93.1 ± 13.3	98.3 ± 14.7	105.0 ± 14.6	99.7 ± 13.9	72.5 ± 5.9	73.6 ± 8.3	88.6 ± 10.6	93.6 ± 9.1
Oxidative stress								
Homocysteine (*μ*mol/L)	12.7 ± 0.9	11.6 ± 1.0	11.6 ± 0.9	11.7 ± 1.0	13.2 ± 0.8^a^	11.5 ± 0.7^b^	10.8 ± 0.9^b^	11.3 ± 0.6^b^
Cysteine (*μ*mol/L)	207.6 ± 8.4^a,b^	195.8 ± 7.7^a^	206.6 ± 7.2^a,b^	219.1 ± 8.9^b^	210.4 ± 9.7^a,b^	186.8 ± 4.9^c^	188.9 ± 7.2^b,c^	220.5 ± 9.9^a^
MDA (*μ*mol/L)	0.9 ± 0.2	1.0 ± 0.2	0.8 ± 0.1	0.8 ± 0.1	1.0 ± 0.1	1.0 ± 0.1	0.8 ± 0.1	0.8 ± 0.1
Antioxidant capacities								
TEAC (*μ*mol/L)	4647.3 ± 129.2	4593.0 ± 109.2	4788.4 ± 139.1	4775.0 ± 132.0	4599.4 ± 79.7^a^	4584.9 ± 124.5^a,b^	4619.2 ± 115.8^a^	5012.7 ± 127.3^b^
SOD (U/mL)	11.3 ± 0.9	11.9 ± 1.2	11.0 ± 1.3	11.5 ± 0.7	12.3 ± 1.0	13.4 ± 1.2	13.6 ± 1.8	13.4 ± 1.8
GSH (*μ*g/mL)	17.9 ± 1.9^a^	11.8 ± 1.1^b^	11.9 ± 1.2^b^	14.2 ± 1.4^b^	17.9 ± 2.4^a^	12.8 ± 2.1^b^	10.1 ± 1.3^b^	11.7 ± 0.9^b^
GSSG (*μ*g/mL)	408.7 ± 19.5	393.2 ± 12.8	407.3 ± 10.7	442.1 ± 15.6	391.5 ± 18.1	385.4 ± 12.0	402.5 ± 7.0	411.2 ± 18.4
GSH/GSSG ratio (×10^−2^)	4.4 ± 1.9	3.0 ± 0.3	3.0 ± 0.4	3.3 ± 0.4	4.7 ± 0.6^a^	3.4 ± 0.6^b^	2.5 ± 0.3^b^	2.7 ± 0.2^b^
GPx (nmol/min/mL)	145.7 ± 20.2^a,b^	117.5 ± 17.0^a^	153.5 ± 29.0^a,b^	169.6 ± 16.8^b^	157.0 ± 19.1	120.6 ± 15.3	136.9 ± 16.5	164.8 ± 20.7
GR (nmol/min/mL)	59.9 ± 2.7	57.1 ± 3.6	59.5 ± 4.5	62.9 ± 4.9	70.7 ± 3.9^a,*∗*^	55.9 ± 3.1^b^	54.4 ± 2.6^b^	54.7 ± 2.7^b^
GST (nmol/min/mL)	40.8 ± 3.5	35.3 ± 3.3	39.5 ± 5.5	37.5 ± 5.7	38.4 ± 4.8	32.4 ± 4.1	45.7 ± 3.7	41.9 ± 4.1

^1^Values are means ± standard error of mean (SE). PLP: pyridoxal 5′-phosphate; BUN: blood urea nitrogen; ALT: alanine aminotransferase; AST: aspartate aminotransferase; ALP: alkaline phosphatase; MDA: malondialdehyde; TEAC: trolox equivalent antioxidant capacity; SOD: superoxide dismutase; GSH: glutathione; GSSG: oxidized glutathione; GPx: glutathione peroxidase; GR: glutathione reductase; GST: glutathione *S*-transferase.

^a,b,c^Values with different superscript letters are significantly different within the group (*p* < 0.05).

^*∗*^Values are significantly different from the placebo group at week 0 (*p* < 0.05).

^*∗∗*^Values are significantly different from the placebo group at week 4 (*p* < 0.05).

^†^Values are significantly different from the placebo group at week 8 (*p* < 0.05).

^††^Values are significantly different from the placebo group at week 12 (*p* < 0.05).
